# Flow cytometry for assessing pulp viability in pediatric dentistry: An exploratory study

**DOI:** 10.34172/joddd.025.44083

**Published:** 2025-12-31

**Authors:** Josué Zuriel Ortiz García, Ismael Secundino, José Luis Ayala Herrera, Alejandro Nava Carmona, Erika Cortes Guzmán, Gloria Ariadna Luevano García, Claudia María Alfaro León, Sandra Liliana Morales Cabrera

**Affiliations:** ^1^Research Department, Faculty of Dentistry, La Salle Bajío University, León, Guanajuato, Mexico; ^2^Department of Emergencies, Faculty of Dentistry, La Salle Bajío University, León, Guanajuato, Mexico; ^3^Department of Pediatric Dentistry, Faculty of Dentistry, La Salle Bajío University, León, Guanajuato, Mexico

**Keywords:** Biomarkers, Flow cytometry, Pediatric dentistry, Pulp viability, Pulpal pathology, Proteomics

## Abstract

**Background.:**

Dental pulp (DP) in pediatric patients plays a vital role in dentin formation, nutrition, protection, and tooth repair. Pulpal pathologies are a common reason for pediatric dental visits; however, accurate clinical diagnosis can be challenging, particularly in young patients and those with limited cooperation. Currently, no reliable clinical method exists to definitively determine pulp viability, and histopathological diagnosis remains the gold standard despite its invasiveness. Flow cytometry has proven effective in cellular analysis across biomedical fields and was used in this study to assess pulp viability in pediatric patients with various pulpal conditions.

**Methods.:**

Forty-four pulp samples from pediatric patients aged 2–7 years were classified as healthy pulp (HP), reversible pulpitis (RP), irreversible pulpitis (IP), and pulp necrosis (PN). Flow cytometry using 7-aminoactinomycin D (7-AAD) staining was employed to assess cell viability. Total protein extracts were obtained from each group for SDS-PAGE analysis of protein profiles. Proteolytic activity was evaluated through gelatin zymography to detect matrix metalloproteinase (MMP) activity.

**Results.:**

Flow cytometry effectively quantified viable cells across diagnostic categories, revealing minimal differences in viability between HP, RP, and IP, which may explain the clinical challenge of differentiating these conditions. Protein profile analysis showed a progressive reduction in the number of protein bands as pulpal disease advanced, although some bands remained consistent. Proteolytic activity, likely associated with MMP-2, increased with disease progression and was significantly elevated in PN compared to other groups.

**Conclusion.:**

Flow cytometry proved a valuable tool for quantifying pulp cell viability in pediatric patients, highlighting the narrow clinical distinction between pulpal conditions. Protein profiling suggests the potential to identify diagnostic biomarkers that support less invasive diagnostic approaches. Although matrix metalloproteinase-2 (MMP-2) activity was present in all stages of pulpal disease, it could not be confirmed as a specific biomarker for disease progression.

## Graphical Abstract

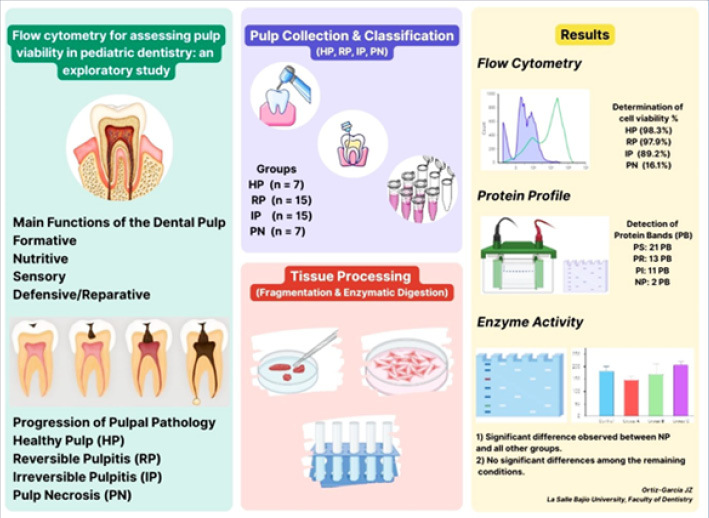

## Introduction

 Dental pulp (DP) is composed of highly innervated and vascularized connective tissue. Its main functions include dentin formation, nutritional support, protection, and tooth repair. Resident cells in the DP include odontoblasts, fibroblasts, macrophages, dendritic cells, lymphocytes, mast cells, and undifferentiatedmesenchymal stem cells.^1,2^ Maintaining DP in optimal conditions is essential toenhance the mechanical resistance of teeth, support physiological tooth eruptionwithout affecting the permanent tooth germ, and prevent both local and systemic orthopedic issues during childhood growth and development.^2,3^ DP lesions may originate from various causes, including infectious processes, trauma, thermal changes, extensive restorations, toxicity from restorative materials, iatrogenic damage, electrogalvanism, altered blood flow, radiation, endogenous intoxications linked to systemic diseases, as well as physiological and idiopathic conditions.^4^ Any of these factors may lead to a condition known as pulpitis.^5^ The American Academy of Pediatric Dentistry (AAPD) classifies pulp conditions into several categories: healthy pulp (HP), which is asymptomatic and responds normally to vitality tests; reversible pulpitis (RP), which is a reversible inflammatory state; irreversible pulpitis (IP), where the pulp is inflamed and unable to return to a healthy state; and pulp necrosis (PN).^6^ These criteria are consistent with those proposed by the American Association of Endodontists (AAE) for the diagnosis of pulpal diseases.^2^ Pulpal lesions are considered one of the main reasons for dental consultations inpediatric patients. However, diagnosis may be complicated by factors such as the patient’s age, cooperation, or systemic condition, which can lead to incorrect treatment planning. To avoid this, several diagnostic and therapeutic methodologies have been proposed, including clinical and radiographic examination, along with pulp vitality assessments using mechanical, sensory, and vitality tests. These evaluations determine the diagnosis and treatment plan for primary teeth,^7^ which may include indirect pulp capping or pulpotomy in cases of RP, or pulpectomy or extraction in cases of IP or PN.^5,7^ Currently, no clinical diagnostic method can accurately determine pulp viability or monitor disease progression, except for histopathological diagnosis,^8^ which requires complete tissue removal and is invasive and complex.In recent decades, flow cytometry has been effectively used as a diagnostic and research tool in various biomedical fields, enabling the characterization of different cell populations from both healthy and pathological tissues.^9^ Therefore, the primary objective of this study was to assess the cellular viability of pulp tissuesobtained from pediatric patients with different clinical diagnoses and pulp treatments using flow cytometry.

## Methods

###  Sample Collection

**Figure 1:**
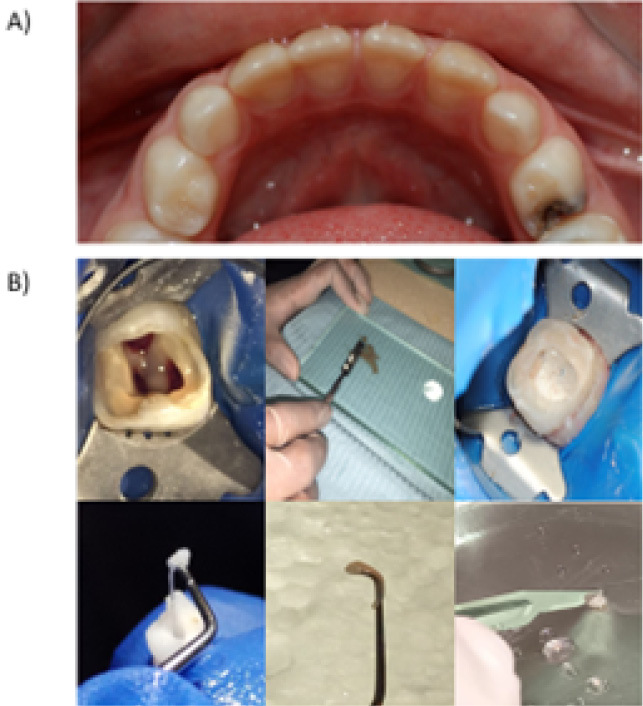
Clinical evaluation of the patients. (A) To allocate participants into study groups, each patient underwent a comprehensive examination and diagnosis by specialists from the Master’s Program in Pediatric Dentistry at La Salle Bajío University. (B) Following pulpal diagnosis, tissue samples were collected in accordance with the indicated clinical procedure: pulpotomy, pulpectomy, or surgical extraction. The standard clinical approach and restorative protocols were strictly adhered to and not altered at any point.

 This study included 44 pulp samples from pediatric patients aged 2–7 years, with a mean age of 4 years. The extracted teeth were primarily molars and were classified into four diagnostic groups: control group: healthy pulp (HP, n = 7), group 1: reversible pulpitis (RP, n = 15), group 2: irreversible pulpitis (IP, n = 15), and group 3: pulp necrosis (PN, n = 7) (Table 1). All the patients were classified as ASA I (American Society of Anesthesiologists Physical Status I). The specimens were provided by the Pediatric Dentistry Master’s Program at the School of Dentistry, Universidad La Salle Bajío, León, Guanajuato, Mexico (Figure 1). The harvested pulp tissues were rinsed with phosphate-buffered saline (PBS) (Sigma-Aldrich) and subsequently placed in Roswell Park Memorial Institute medium (RPMI) medium (Sigma-Aldrich) supplemented with 100-μg/mL streptomycin (PISA). Ten pulp samples were processed immediately for cell isolation and viability analysis by flow cytometry. From the remaining samples, total protein extracts were obtained to analyze protein expression profiles for each diagnosis and to assess tissue degradation capacity using polyacrylamide gel zymography. In all cases, HP samples were used as the baseline control. This study complied with the Declaration of Helsinki for medical research protocols and was reviewed and approved by the Ethics Committee of Universidad La Salle Bajío, León, Guanajuato, Mexico, under the registration number CEIS 0018-300924.

**Table 1:** Pulp tissues analyzed in the study

**Diagnostic groups**	**Female**	**Male**	**Treatment**
Healthy pulp (n = 7)	3	4	Indicated surgical extraction
Reversible pulpitis (n = 15)	6	9	Pulpotomy
Irreversible pulpitis (n = 15)	9	6	Pulpectomy
Pulp necrosis (n = 7)	3	4	Pulpectomy / Extraction

###  Flow Cytometry 

 To isolate cells, 10 pulp samples were cut into small fragments using a #15 scalpel blade and incubated in 1 mL of collagenase (Sigma-Aldrich) at 37°C for 1 hour. After incubation, the samples were washed with 1 mL of PBS and filtered through a cell strainer membrane. The resulting cells were collected and centrifuged at 2450 × *g* for 10 minutes. They were then resuspended in 1 mL of PBS and counted using a Neubauer chamber. For the viability assays, 10,000 cells were taken from each diagnostic group (Table 2) and incubated for 15 minutes with the fluorochrome 7-aminoactinomycin D (7-AAD) (Thermo Fisher Scientific), which is primarily used to assess cell viability.^10^ The samples were then analyzed using a flow cytometer (BD Accuri C6 Plus) with an excitation wavelength of 488 nm.

**Table 2:** Distribution of study groups

**Group**	**Pulp condition **	**Fluorochrome**
Control	Healthy pulp	7-AAD
Group 1	Reversible pulpitis	7-AAD
Group 2	Irreversible pulpitis	7-AAD
Group 3	Pulp necrosis	7-AAD

7-AAD: 7-aminoactinomycin D.

###  Protein Extraction 

 Protein extraction was performed as described by Vincourt et al.,^11^ with minormodifications. This procedure was carried out either in the absence (for gelatin zymography) or presence (for protein profiling) of a protease inhibitor cocktail (Roche Complete, Sigma-Aldrich). The proteins were precipitated with trichloroacetic acid (TCA)-acetone and resuspended in a rehydration solution containing 7-M urea, 2-M thiourea, 2% 3-[(3 cholamidopropyl)dimethylammonio]-1-propanesulfonate (CHAPS), 0.5% immobilized pH gradient (IPG) buffer, and 0.1% bromophenol blue, supplemented with 2-mM dithiothreitol (DTT). For zymography gels, the proteins were resuspended in homogenization buffer (50-mM Tris-HCl, 0.5% Triton X-100, pH = 7.4). Protein concentration was determined using the 2D Quant Kit (Amersham, GE Healthcare).

###  Protein Profiles by SDS-PAGE

 From the pulp samples of each study group (Table 2), total protein extracts were obtained and pooled by diagnosis. A total of 30 µg of protein per group was mixed with Laemmli 2X buffer (v/v) and separated by 10% SDS-PAGE. The gels were stained overnight with Coomassie Brilliant Blue (Sigma-Aldrich) and destained using a solution of 30% methanol and 10% acetic acid. Finally, the gels were stored in distilled water for preservation and subsequent photodocumentation.

###  Gelatin Zymography

 Protein samples (30 µg) were mixed with Laemmli 2X buffer (without 2-mercaptoethanol) (v/v) and separated on 8% SDS-PAGE gels co-polymerized with 1% gelatin (Sigma-Aldrich). The gels were then incubated in activation buffer (50-mM Tris-HCl, pH = 7.4, 5-mM CaCl₂, and 1-μM ZnCl₂) for 48 hours at 37°C. After incubation, the gels were stained with Coomassie Blue and destained with a solution of 30% ethanol and 10% acetic acid. Proteolytic activity was visualized as clear bands against a blue background.

###  Statistical Analysis

 For quantitative analysis, proteolytic activity quantified by densitometry was compared among the four diagnostic groups using a one-way analysis of variance (ANOVA). When significant differences were detected, post hoc Tukey tests were applied for pairwise comparisons. Statistical significance was set at *P* < 0.05. Analyses were performed using GraphPad Prism version 10.3.1. Exact *P*-values for significant comparisons are reported in the Results section.

## Results

###  Determination of Cell Viability in HP, RP, IP, and PN

**Figure 2:**
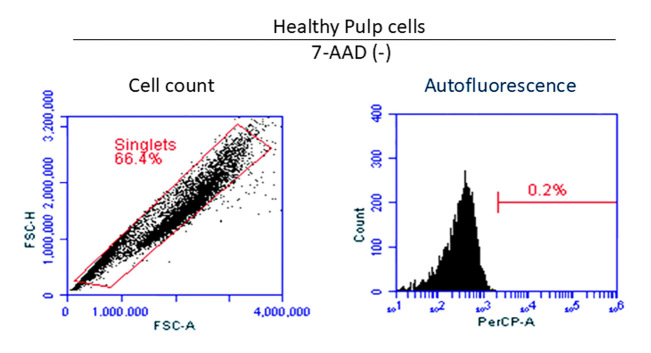
Determination of autofluorescence. (A) The total cell count of the control group, where cells of various sizes and complexity can be observed. (B) After analyzing the cells without 7-AAD, it was determined that only 20 out of 10,000 cells emitted autofluorescence, demonstrating that this result does not interfere with the final analysis.

**Figure 3:**
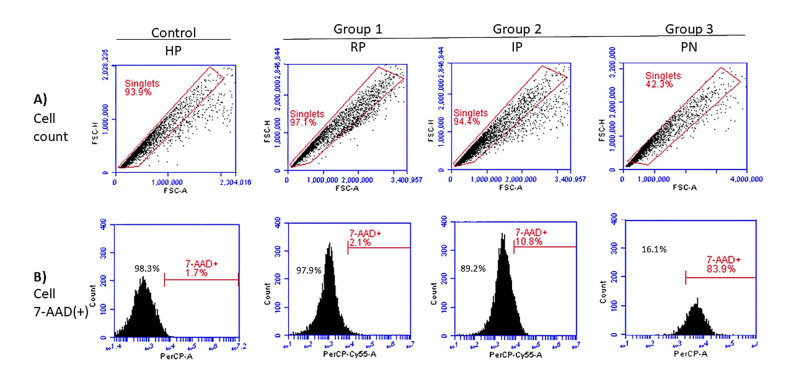
Evaluation of cell viability. (A) The panels corresponding to each analyzed group, where the total cell count per reading was determined. Cellular populations of varying sizes and complexity are also observed. (B) Representative histograms of the events evaluated in the different stages of pulpal pathology are shown. As the pulpal lesion progresses, an increase in 7-AAD fluorescence is observed, suggesting a decrease in cell viability.

 To evaluate the autofluorescence emitted by pulp tissue cells, the group control HP was analyzed without incubation with the fluorochrome 7-AAD to determine whether the autofluorescence emitted by each analyzed event would interfere with the final result (Figure 2). Subsequently, each of the study groups (RP, IP, and PN) was analyzed in the presence of the 7-AAD fluorochrome, and the percentage of cell viability was determined for each case (Figure 3) (Table 3).

**Table 3:** Determination of cell viability per 10,000 events analyzed

**Group **	**Pulp condition **	**7-AAD+(%) **	**Viable cell**
Control	Healthy pulp	1.7	9830 (98.3%)
Group 1	Reversible pulpitis	2.1	9790 (97.9%)
Group 2	Irreversible pulpitis	10.8	8920 (89.2%)
Group 3	Pulp necrosis	83.9	1610 (16.1%)

7-AAD: 7-aminoactinomycin D.

###  Evaluation of Protein Profiles

**Figure 4:**
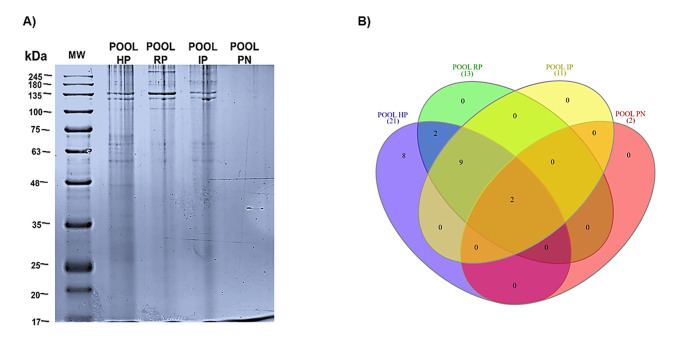
SDS-PAGE. (A) Proteins from each pulpal diagnosis were separated within a molecular weight range of 245 kDa to 17 kDa. As pulpal disease progressed, the visibility of protein bands (pb) gradually decreased (HP: 21 pb, RP: 13 pb, IP: 11 pb, PN: 2 pb). (B) Using the InteractiVenn program, we analyzed protein band interactions across the four pulpal conditions evaluated. Although all conditions shared protein bands, it is noteworthy that 8 protein bands were exclusive to the HP condition, and 2 protein bands remained constant throughout the progression of pulpal disease. This suggests that these may be constitutive proteins of the dental pulp and could play an important role at each stage of pulpal pathology. Protein bands were classified according to their molecular weight to facilitate the interaction analysis.

 To achieve a higher protein concentration, a pooled protein extract was prepared from the samples corresponding to each diagnosis. Subsequently, 30 µg of protein from each group was separated using 10% SDS-PAGE and stained with Coomassie blue. It was observed that as pulpal disease progressed, the number of protein bands decreased noticeably (Figure 4A). Conversely, some protein bands were consistently present throughout the progression of pulpal pathology (Figure 4B).

###  Proteolytic Activity

**Figure 5:**
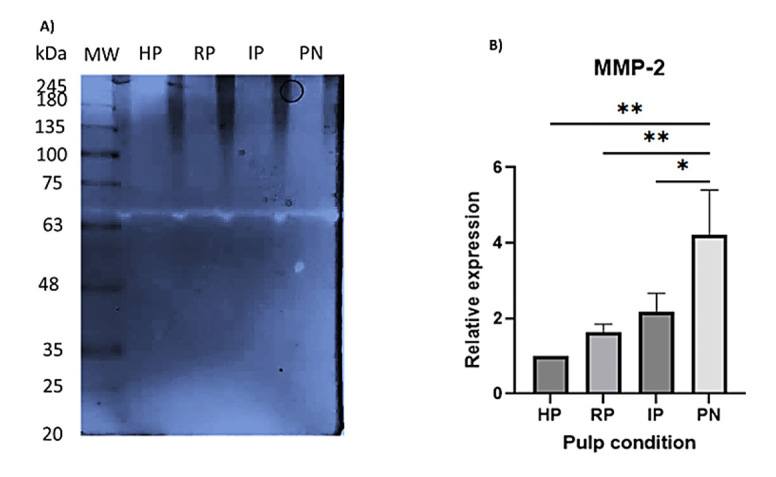
Proteolytic activity in pulp samples. (A) Representative gelatin zymogram showing a proteolytic band at an approximate molecular weight of 63 kDa across all groups (HP, RP, IP, and PN). The intensity of this band increased with the severity of the pulpal condition. (B) Densitometric quantification of the 63 kDa band. Data are expressed as mean values, and error bars indicate the standard error. HP was arbitrarily set to 1 for normalization. The groups were statistically analyzed using one-way ANOVA, revealing significant differences in the following comparisons: HP vs. PN (**P = 0.0013), RP vs. PN (**P = 0.0053), IP vs. PN (*P = 0.0204)

 Gelatin zymography revealed a proteolytic band of approximately 63 kDa in all diagnostic groups, with increasing intensity according to disease severity (Figure 5A). This band is consistent with the expected molecular weight of matrix metalloproteinase-2 (MMP-2). Densitometric analysis confirmed a progressive increase in MMP-2 activity, with the PN showing significantly higher proteolytic activity compared with all other groups (HP vs. PN, *P* = 0.0013; RP vs. PN, *P* = 0.0053; IP vs. PN, *P* = 0.0204). No statistically significant differences were observed between HP, RP, and IP (Figure 5B).

## Discussion

 The development of pulpal pathologies in childhood is primarily associated with carious processes,^8,12^ which, if left untreated, can lead to the loss of affected teeth. Traditionally, pulpal diagnosis has relied on clinical and radiographic assessments;^6,8,13^ however, since the biological and molecular mechanisms underlying pulpal disease are not yet fully understood, accurately determining pulp viability when it becomes compromised remains challenging.^2^ This limitation underscores the need for objective biological biomarkers that can support more precise and reliable diagnostic decision-making. Despite efforts to correlate dental pulp viability with its clinical and histopathological characteristics, this relationship remains uncertain in clinical practice,^8,14,15^ due to the complexity of the microenvironment that develops during the progression of pulpal pathology. Currently, flow cytometry has become an essential tool for research and diagnostics in the biomedical field.^9,16^ In dentistry, it has been used to characterize cellular populations of the dental pulp, among which dental pulp stem cells are particularly notable.^17,18^ To the best of our knowledge, this is the first study to evaluate pulp viability in pediatric patients using flow cytometry across the different stages of pulpal pathology. This approach provides objective evidence of the cellular status of the pulp under real clinical conditions and opens the door to the development of quantitative and molecular diagnostic tools in pediatric dentistry. The relevance of these findings lies in the fact that pulp viability was assessed at the time of pulp extirpation and correlated with the clinical diagnosis. We observed a very narrow difference in the percentage of viable cells between HP, RP, and IP. These results may help explain why pulpal diagnosis can be a complex procedure, particularly for clinicians with limited experience in pediatric pulpal diagnostics, and may also clarify the reasons behind the failure of certain clinical treatments. Furthermore, although the differences in cell viability between HP, RP, and IP were minimal, these findings carry important clinical implications. The similarity in the percentage of viable cells suggests that early or moderate inflammatory changes do not necessarily lead to a substantial loss of cellular viability. This may help explain why conventional diagnostic tests lack precision in distinguishing between contiguous clinical states, contributing to ambiguous diagnoses and, in some cases, suboptimal therapeutic outcomes. Our data indicate that cell viability alone may not be a sufficiently sensitive indicator to differentiate early stages of pulpal pathology, but it opens the door to the search for new diagnostic tools within the field of molecular biomarkers. Several authors have attempted to decipher the proteome present in saliva and healthy dental tissues,^19-21^ as well as in carious lesions,^22,23^ odontogenic cysts and tumors,^24,25^ and in various pulpal conditions,^17,19,26-29^ to understand the role of expressed proteins during the development and progression of these pathologies and propose possible diagnostic biomarkers to improve therapeutic plans for each pathological entity. In particular, Silva et al.,^30^ Loureiro et al.,^31^ and Yue et al.^32^ have identified protein profiles expressed in healthy, inflamed, and necrotic pulps, each with specific proteins. These findings may help better understand the mechanisms underlying the microenvironment and the evolution of pulpal pathology. In our protein profiles, we observed that as pulpal disease progressed, the protein band pattern changed with each pathological condition. This suggests that specific biomarkers could be identified for each stage of pulpal disease progression, thereby providing diagnostic aids to improve treatment plans in deciduous and immature permanent teeth. Considering that pulpal disease is progressive and invasive, we evaluated proteolytic activity at each stage through zymography assays, since metalloproteinases have been shown to play a crucial role in both physiological and pathological conditions, at systemic and oral levels.^33-35^ It is noteworthy that an increase in proteolytic activity was observed as the disease progressed. However, no statistically significant differences were found between HP, RP, and IP, unlike PN, which showed significant differences when compared to the previous conditions. Finally, it is important to mention that this study had several limitations that should be considered when interpreting the results, such as the relatively limited sample size, particularly in the HP and PN groups, which may restrict the generalization of the findings. In addition, the protein extracts for SDS-PAGE and zymography were obtained from pooled samples within each diagnostic category, which prevents the assessment of individual variability and could underestimate biologically relevant differences. Future studies with larger sample sizes and individual analyses will be necessary to validate and expand these results, and to continue deepening our understanding of pulp viability.

## Conclusion

 Using flow cytometry, we quantified the percentage of viable cells at each stage of pulpal pathology in pediatric patients, highlighting the narrow margin that exists between stages. This, combined with the individual conditions of each patient, poses a challenge to accurate pulpal diagnosis. Furthermore, understanding the behavior of protein profiles at each stage of pulpal pathology provides an opportunity to identify biomarkers in pulpal lesions and develop less invasive diagnostic methods for pediatric patients. Finally, although we identified proteolytic activity at every stage of pulpal pathology, our data suggest that MMP-2 cannot be considered a biomarker for this condition.

## Competing Interests

 The authors declare no conflict of interest in this study.

## Ethical Approval

 Ethics Committee of Universidad La Salle Bajío (CEIS 0018-300924).
